# Animal models of *Mycobacterium abscessus* pulmonary infection phenotypes: What are we modeling?

**DOI:** 10.1371/journal.ppat.1013414

**Published:** 2025-08-11

**Authors:** Xiangyu Sui, Stefan H. Oehlers

**Affiliations:** 1 A*STAR Infectious Diseases Labs (A*STAR ID Labs), Agency for Science, Technology and Research (A*STAR), Singapore, Singapore; 2 Department of Microbiology and Immunology, Yong Loo Lin School of Medicine, National University of Singapore, Singapore, Singapore; University of Geneva: Universite de Geneve, SWITZERLAND

Pulmonary infections caused by *Mycobacterium abscessus* have been difficult to model as most wild-type mice rapidly clear infection across a range of inoculation routes. Existing animal models of *M. abscessus* infection can be broadly classified into acute overwhelming infection, self-limiting mucosal clearance, and chronic granulomatous pathologies. We will make the argument that each of these models potentially models distinct endotypes of *M. abscessus* infection and highlight opportunities to understand infection biology through comparison of experimental models.

## 1. Is *M. abscessus* molecular pathogenesis just TB with a different mycobacterial species?

*Mycobacterium tuberculosis* is the most successful human pathogen having claimed more lives than any other species across history and remains the number one microbial species responsible for infectious disease deaths globally. The genus level similarities between *M. abscessus* and *M. tuberculosis* span genomic similarity demonstrated by both genetic measures such as sequence similarity and essential gene analyses, and pathogenic manifestations such as chronic pulmonary infection and residency within macrophages [1,2]. However, the primary *M. tuberculosis* virulence factor is the early secretory antigenic target secretion system-1 (ESX-1) while *M. abscessus* lacks ESX-1 but has two analogous type VII secretion systems ESX-3 and ESX-4 which are necessary for *in vivo* virulence [3]. Even with relatively few isolates of *M. abscessus* sequenced, it is clear that the *M. abscessus* genome is highly diverse with signs of active horizontal gene transfer compared to *M. tuberculosis* [4].

On the host side, shared susceptibility factors include Mendelian susceptibility to mycobacterial disease (MSMD) genes and autoantibodies impairing IFN-γ signaling, and immunosuppression by inhaled corticosteroids [5–8]. Interestingly, divergent host susceptibility factors also provide evidence of unique molecular pathogenesis. Cystic Fibrosis (CF) and Hyper Immunoglobulin E Syndrome are two examples where increased susceptibility to mycobacterial infection is seen for *M. abscessus* but not *M. tuberculosis* [9–11]. While HIV/AIDS is a well-known risk factor for *M. tuberculosis* infection, there is limited evidence for increased susceptibility to *M. abscessus* in this population [12], although we must note that the availability of effective anti-retroviral therapy over the last 20+ years may mask any association. These distinctions, along with the observation that *M. abscessus* infection more commonly occurs in individuals with underlying structural lung diseases such as non-CF bronchiectasis, highlights the divergent host factors influencing susceptibility [13].

## 2. What do we know about the host–pathogen interface of *M. abscessus* infection?

Unfortunately, the answer is not much. Clinical specimens are mainly microbiological isolates and host peripheral blood rather than *in situ* biopsy samples of *M. abscessus* at the host-pathogen interface. The paucity of *M. abscessus* biopsy and autopsy samples continues to limit our understanding of *M. abscessus* within-host molecular diversity and adaptation, and host immune responses at the sites of *M. abscessus* infection.

Previous research has proposed that *M. abscessus* nodular disease initiates from patients being colonized by a smooth colony morphotype from the environment, which accumulates mutations in the genotoxic host environment from antibiotic and immune sources, causing an irreversible transition to a rough colony morphotype lacking glycopeptidolipids. It is then the exposed surface of rough colony morphotype bacteria that drives excessive host inflammation and tissue destruction that manifests as cavitary disease [13,14]. This chain of events raises the questions of why nodular and cavitary disease manifest in different parts of the lung and if animal models will contribute to an explanation for the spatial distribution of colony morphotypes?

There is strong evidence for the increased experimental virulence and clinical severity of rough morphotype *M. abscessus* from clinical isolates and isogenic strains [14–18]. However, recent large-scale phenotypic profiling suggests that there is more to *M. abscessus* virulence than smooth to rough colony morphotype transition with Boeck and colleagues finding no association between colony morphotype and experimental virulence, clinical clearance or lung function decline across 199 diverse clinical isolates [19]. One limitation of this study was the lack of link between morphotype and radiologic diagnosis to allow correlation of experimental data to disease endotype.

Indirect observation of the host-pathogen interface through peripheral blood analysis identifies core immune responses to mycoacterial infection, such as the production of the ubiquitous cytokine interferon gamma. A potentially important differential is higher levels of IL-17 in *M. abscessus* infection compared to *M. tuberculosis*. This may reflect the relative lack of immune subversion carried out by *M. abscessus* to shape a permissive immune response away from a “default” lung surface damage immune response through active subversion by ESX-1 and Hip1 activities, two factors that are absent in *M. abscessus* [20–22].

## 3. Acute non-resolving infection models uncover cell biology and predict antibiotic activity

Immunocompromised mouse models allow high levels of *M. abscessus* growth in the lungs (recently reviewed in [23]). Despite shortcomings in modeling disease pathobiology, these models provide sufficient *M. abscessus* growth for studying the effect of therapeutics in the context of mammalian pharmacodynamics. The GM-CSF-deficient mouse model is of particular note as it produces strong histopathology and is responsive to chemotherapy.

Systemic infection of zebrafish embryos with *M. abscessus* by intravenous infection is the simplest vertebrate model of *M. abscessus* infection. This model pits *M. abscessus* against the developing innate immune system of the zebrafish embryo and can be titrated, by inoculum and embryo age at the time of infection, to deliver an outcome between overwhelming infection and elimination of the bacteria. This model has utility in examining antibiotic and phage therapeutics [24,25], and the cell biology of host susceptibility to *M. abscessus* infection [10]. However, this model produces little histopathology for correlation to pulmonary disease.

## 4. Self-limiting mucosal infection models reflect aspects of CF and nodular infection

Embedding *M. abscessus* within agar beads and instillation into the lungs of mice causes granulomatous inflammation in the bronchi and small airways [26–28]. The reproduction of non-necrotic granulomatous inflammation and *M. abscessus* replication holds promise for replicating CF pathologies in combination with CFTR-deficient mice [29]. Importantly, bacterial burden and pulmonary pathology are self-limiting with bacterial clearance around 2–3 months post-implantation and pulmonary recovery soon after resorption of the agarose beads in most animals providing a significant window for experimentation.

The role of a pre-existing inflammatory condition in concert with structural lung damage is clear from the predilection of bronchiectasis and COPD patients to *M. abscessus* infection. The βENaC overexpression mouse has pronounced airway inflammation and is susceptible to mucosal surface growth of *M. abscessus* without significant penetration of tissue or progression down to alveoli [30]. Like other immunocompetent mice, infection is self-limiting with bacterial clearance seen by 1 month providing a shorter window for experimentation.

A ciliopathy bronchiectasis mouse model has been described utilizing knockout of IFT88 KO to drive pulmonary inflammation prior to *M. abscessus* infection in agarose beads [31]. This model generates granulomas and maintains bacterial burden beyond lifespan of the beads demonstrating *M. abscessus* engraftment. Notably, the authors found a possible direct role for cilia function in supporting immunity to *M. abscessus*, contributing to the understanding of immunopathology in diseases characterized by impaired ciliary function, such as CF and COPD. Use of this model is limited by a relatively complicated scheme for timed deletion of *IFT88* by Cre/loxP induction and, like the preceding CFTR and βENaC genetic models allows use of high-resolution mouse immunology tools to study the host-pathogen interface.

Multiple instillations of immunocompetent rats with *M. abscessus* agarose beads twice facilitates expansion of *M. abscessus* load after 1 month and granuloma formation without central necrosis [32]. While the distribution of histopathology was not disclosed in the published report, we want to highlight the similarities between these mouse and rat models using agar bead instillation and the nodular bronchiectatic form where *M. abscessus* attacks the upper part of the bronchial tree leading to the tree-in-bud radiologic pattern of infection. We hypothesize that these sites of infection are likely to consist of cellular (non-necrotic) granulomas formed where *M. abscessus* has breached the mucus layer and remains in prolonged contact with mucosal tissue. The development of the rat model is particularly exciting in the field as CF rats have been reported to be spontaneously colonized by the CF-associated pathogen *Pseudomonas aeruginosa* raising the possibility that their respiratory system could be colonized by *M. abscessus* [33].

## 5. Do chronic granulomatous infection models recreate fibrocavitary lesion pathology?

An accessible mouse model of dexamethasone immunosuppression both sides of the infection inoculation yields chronic *M. abscessus* engraftment and a range of titratable granuloma phenotypes following removal of immunosuppression [34]. This is potentially a powerful model of prior corticosteroid usage which places patients at risk of *M. abscessus* for years after treatment cessation [6]. Paradoxically, the damage caused by unchecked *M. abscessus* growth during the immunosuppressive phase may fulfill the requirements of a creating a hyperinflammatory environment and structural lung damage that are missing from the βENaC overexpression mouse.

Adult zebrafish are susceptible to *M. abscessus* infection and produce necrotic granulomas within weeks to months of intraperitoneal infection [35]. The adult zebrafish platform recapitulates the increased pathology associated with rough compared to smooth morphotype *M. abscessus*. Strikingly, knockdown of genes involved in TNF signaling reduced the growth of rough morphology *M. abscessus* and associated pathology suggesting a key divergence in immune containment of rough colony morphotype *M. abscessus* compared to all other mycobacteria, but fits with the clinical observation that tissue damage is immune-mediated.

The fulminant growth of *M. abscessus* in these models and extensive tissue destruction in the face of a competent immune system lead us to hypothesize that these models will most closely resemble cavitary *M. abscessus* infection. We believe cavitary *M. abscessus* infection is preceded by rapid growth of *M. abscessus* either in cavitary pockets or host tissue which forces the formation of granulomas around existing extracellular *M. abscessus*.

### Concluding remarks: The need for comparative host models

Comparative infection biology allows the models discussed here to be hypothetically linked to range of radiological disease classifications. Unlike the extensive molecular and cellular characterization on human TB granulomas, there are almost no characterizations of *M. abscessus*-infected human tissue in the literature. Rather than settling on a single experimental platform, we propose that multiple *M. abscessus* infection models will be required to answer the key outstanding questions in *M. abscessus* pathogenesis (Table 1).

**Table 1:** Key challenges.

Topic	Challenge
Human susceptibility	Delineate the link between prior TB and *Mycobacterium abscessus* susceptibility. Is there a shared genetic/environmental susceptibility profile for these species of mycobacteria? Can genetically diverse mouse panels untangle conserved and divergent host susceptibility factors to TB and *M. abscessus* infections? [36]
Bacterial diversity	Life beyond ATCC19977 and the rough vs smooth dichotomy. What is the molecular basis for Asian strains associated with fibrocavitary pathology? How prevalent are “phenotypic rough” cell surfaces in vivo?
Acute infection models	Reproduce the biofilm and granulomatous growth modes that shield mycobacteria from antibiotics.
Mucosal infection models	How do competitive interactions with other opportunistic pathogens drive lung disease in vivo?
Granulomatous infection models	Mapping experimental pathology to nodular or fibrocavitary clinical pathologies.
